# Is there a neuroanatomical basis of the vulnerability to suicidal behavior? A coordinate-based meta-analysis of structural and functional MRI studies

**DOI:** 10.3389/fnhum.2014.00824

**Published:** 2014-10-22

**Authors:** Kees van Heeringen, Stijn Bijttebier, Stefanie Desmyter, Myriam Vervaet, Chris Baeken

**Affiliations:** Unit for Suicide Research, Department of Psychiatry and Medical Psychology, Ghent UniversityGhent, Belgium

**Keywords:** suicide, vulnerability, meta-analysis, gray matter, decision-making

## Abstract

**Objective:** We conducted meta-analyses of functional and structural neuroimaging studies comparing adolescent and adult individuals with a history of suicidal behavior and a psychiatric disorder to psychiatric controls in order to objectify changes in brain structure and function in association with a vulnerability to suicidal behavior.

**Methods**: Magnetic resonance imaging studies published up to July 2013 investigating structural or functional brain correlates of suicidal behavior were identified through computerized and manual literature searches. Activation foci from 12 studies encompassing 475 individuals, i.e., 213 suicide attempters and 262 psychiatric controls were subjected to meta-analytical study using anatomic or activation likelihood estimation (ALE).

**Result:** Activation likelihood estimation revealed structural deficits and functional changes in association with a history of suicidal behavior. Structural findings included reduced volumes of the rectal gyrus, superior temporal gyrus and caudate nucleus. Functional differences between study groups included an increased reactivity of the anterior and posterior cingulate cortices.

**Discussion**: A history of suicidal behavior appears to be associated with (probably interrelated) structural deficits and functional overactivation in brain areas, which contribute to a decision-making network. The findings suggest that a vulnerability to suicidal behavior can be defined in terms of a reduced motivational control over the intentional behavioral reaction to salient negative stimuli.

## INTRODUCTION

It is estimated that one million people commit suicide annually (World Health Organisation [WHO], 2002). Non-fatal suicidal behavior occurs 10–20 times more frequently, and a history of self-harm or suicide attempts is the strongest risk factor for suicide, being present in at least 40% of cases (Hawton and van Heeringen, 2009). Human and societal costs of suicidal behavior are substantial, and many countries have recently developed suicide prevention programs. The prevention of suicide, however, still poses major challenges. Clinicians are unable to predict the occurrence of suicidal behavior. In addition, when suicide risk is considered high, its management proves challenging because of the poor evidence base. It is, for example, impossible to predict whether an individual at risk will respond to treatment with a decrease or an increase in suicide risk. Even if there is a positive response to treatment, it is often unclear how and why this happened. Suicide prevention is thus in great need of markers that predict suicidal behavior and serve as a substrate for treatment.

Based upon the current state of knowledge, a stress-diathesis or stress-vulnerability model of suicidal behavior has been developed (Van Heeringen, 2012). In general, stress-vulnerability models describe the mechanisms due to which diathetic or vulnerable individuals respond with abnormal or pathological reactions to physiological stimuli or the ordinary conditions of life that are borne by the majority of individuals without injury (Zuckerman, 1999). With regard to suicidal behavior, stressors including interpersonal, professional, or financial problems commonly precipitate these behaviors. In addition, suicidal behavior occurs, in general, in the context of psychiatric conditions such as depression or bipolar disorder. However, only a small proportion of individuals confronted with stressors and suffering from these disorders will actually show suicidal behavior. The stress-vulnerability model of suicidal behavior may thus explain such behavior as the consequence of an interaction between exposure to stressors and a vulnerability in individuals suffering from psychiatric disorders. Comparing individuals with a history of a psychiatric disorder and suicidal behavior to those with a history of such a psychiatric disorder but no history of suicidal behavior can thus be expected to shed light on the particular vulnerability to suicidal behavior.

Evidence of a neurobiological basis of the vulnerability to suicidal behavior is increasing (van Heeringen and Mann, 2014). From a neurochemical point of view, the serotonin neurotransmission system and the HPA-stress-response system appear to be crucially involved. At a neuroanatomical level, postmortem and neuroimaging studies show changes in a number of areas in the brain in association with a vulnerability to suicidal behavior. Four reviews of neuroimaging studies have recently been published (Desmyter et al., 2011; Jollant et al., 2011; van Heeringen et al., 2011; Zhang et al., 2014). These reviews agreed on the involvement of particular brain areas in the development of suicidal behavior, including the dorsolateral and orbitofrontal cortex. However, no clear picture emerged from the reviews with regard to other cortical regions and subcortical involvement. Moreover, sample sizes of studied patient groups were small, which hampers the interpretation of findings.

In order to investigate whether the subjective conclusions from the reviews can be objectified, and, more importantly, to specifically study the vulnerability to suicidal behavior using neuroimaging, we performed coordinate-based meta-analyses of structural and functional neuroimaging studies (CBMA; Turkeltaub et al., 2002). The objective of CBMA is not to estimate the magnitude of an effect across studies, but rather to identify anatomical locations in which an effect is observed consistently. By including only studies in which brain structure or function of individuals with a history of a psychiatric disorder and suicidal behavior were compared to brain structure or function of those with a history of the psychiatric disorder but no suicidal behavior, this meta-analysis aimed at investigating particularly the association between a vulnerability to suicidal behavior and brain structure or function.

## MATERIALS AND METHODS

### DATA SEARCH

A search was carried out using Web of Science, PsycINFO, and Pubmed databases with the keywords: suicide, suicidal, neuroimaging, magnetic resonance imaging (MRI), positron emission tomography (PET), single photon emission tomography (SPET), single photon emission computerized tomography (SPECT), voxel based morphometry (VBM). Criteria for including studies in the meta-analyses were:

(1) Peer-reviewed original structural or functional brain imaging study published up to July 2013.
(2) Use of PET, SPE(C)T or MRI.
(3) Contrast between individuals with a history of a psychiatric disorder and suicidal behavior and individuals with a history of the psychiatric disorder but no suicidal behavior
(4) Adolescents and adults.
(5) Results reported as coordinates in a normalized standard stereotactic space, i.e., the Talairach or Montreal Neurological Institute (MNI) reference system.

Unpublished studies, case reports or conference abstracts were not included, as were studies reporting only region-of-interest findings or using seed-voxel-based analysis procedures. Studies comparing elderly suicide attempters to elderly psychiatric controls were not included in the meta-analysis, because the neurobiology of suicidal behavior may differ between the elderly and adolescents or adults. The reference lists of relevant papers were checked manually for additional relevant publications not previously identified.

The literature search identified articles reporting on 22 structural and 16 functional brain imaging studies, in which individuals with a history of suicide attempts were included and compared to controls.

Seven structural imaging studies were excluded because Talairach or MNI coordinates of identified anatomical structures were not reported (Monkul et al., 2007; Matsuo et al., 2010; Vang et al., 2010; Cyprien et al., 2011; Spoletini et al., 2011; Nery-Fernandes et al., 2012; Giakoumatos et al., 2013). Two studies were excluded because the study population consisted of elderly (Hwang et al., 2010; Dombrovski et al., 2012). Two additional studies were excluded because results for suicide attempters were not reported separately as attempters were regarded as part of a larger group of individuals considered at increased risk of suicide (Wagner et al., 2011, 2012). Finally, five studies were excluded from the meta-analysis because they focused specifically on hyperintensities, i.e., white matter hyperintensities (Ahearn et al., 2001) or gray matter hyperintensities (Ehrlich et al., 2004, 2005; Pompili et al., 2007, 2008).

Identified functional imaging studies used SPECT (*n* = 4; Audenaert et al., 2001, 2002; Lindstrom et al., 2004; Ryding et al., 2006), PET (*n* = 4; Meyer et al., 2003; Oquendo et al., 2003; Leyton et al., 2006; Nye et al., 2013), and fMRI (*n* = 8; Jollant et al., 2008, 2010; Pan et al., 2011, 2013a,b; Marchand et al., 2012; Dombrovski et al., 2013; Fan et al., 2013). A number of studies had to be excluded from the meta-analysis due to absence of psychiatric controls (Audenaert et al., 2001, 2002; Oquendo et al., 2003; Leyton et al., 2006), or the absence of stereotactic coordinates in the report (Meyer et al., 2003; Lindstrom et al., 2004; Ryding et al., 2006; Nye et al., 2013). One study was not included because the analyses focused only on striatal and cortical midline structures (Marchand et al., 2012), while another study was excluded as the study population consisted of elderly individuals (Dombrovski et al., 2013).

### ALE META-ANALYSIS

Reported coordinates were analyzed for topographic convergence using the revised ALE algorithm for coordinate-based meta-analysis of neuroimaging results (Turkeltaub et al., 2002; Eickhoff et al., 2009). The goal of coordinate-based meta-analyses of neuroimaging data is to identify brain areas where the reported foci of activation converge across published experiments. To this end, the meta-analysis determines if the clustering is significantly higher than expected under the null distribution of a random spatial association of results from the considered experiments while acknowledging the spatial uncertainty associated with neuroimaging foci. As the first step, and following conversion from MNI space to Talairach space using icbm2tal where needed, reported foci were interpreted as centers for 3D Gaussian probability distributions that capture the spatial uncertainty associated with each focus. This uncertainty is mostly a function of between-template (attributable to different normalization strategies and templates across laboratories) and between-subject (due to small sample sizes) variance. In fact, the between-template and between-subject variability are acknowledged based on empirical estimates, the latter being additionally gaged by individual sample size (Eickhoff et al., 2009). In a second step, the probabilities of all activation foci in a certain experiment were combined for each voxel, yielding a modeled activation (MA) map (Turkeltaub et al., 2012). Voxel-wise ALE scores resulted from the union across these MA maps and quantified the convergence across experiments at each particular location in the brain. The third and last step distinguished between random and “true” convergence by comparing the ensuing ALE scores against an empirical null distribution reflecting a random spatial association between the experiments’ MA maps (Eickhoff et al., 2012). The within-experiment distribution of foci, however, was regarded as fixed (Eickhoff et al., 2009). Thus, a random-effects inference was invoked, focusing on the above-chance convergence across different experiments (Eickhoff et al., 2009; Caspers et al., 2010; Kurth et al., 2010). The resulting ALE scores were tested against the earlier calculated “true” ALE scores and cut off at a cluster-level-corrected threshold of *p* < 0.05. The cluster size threshold was >200 mm^3^.

## RESULTS

### STRUCTURAL IMAGING STUDIES

As shown in **Table 1**, six structural imaging studies were included in the meta-analysis (Aguilar et al., 2008; Rusch et al., 2008; Jia et al., 2010; Benedetti et al., 2011; Mahon et al., 2012; Soloff et al., 2012).

**Table 1:** Structural imaging studies meeting inclusion criteria.

Study (first author)	Year	Imaging modality	Study population SA/PC/HC	Mean age years	% *F*	Measurement	Available data
Aguilar	2008	MRI 1,5T	S 13/24/0	37/43/0	0/0/0	VBM	BA; *x, y, z*; T; *p*
Rüsch	2008	MRI 1,5T	S 10/45/55	30/37/36	30/40/38	VBM	*x, y, z*; *p*; absolute volume (IU); F; *p*
Jia	2010	MRI 3T	MD 16/36/52	34/35/37	69/44/54	DTI FA	*x, y, z*; cluster size; F; t
Benedetti	2011	MRI 3T	BD 19/38/0	44/46/0	56/72/0	VBM	BA; *x, y, z*; cluster size; Z; *p*
Mahon	2012	MRI 1,5T	BD 14/15/15	33/37/34	36/40/47	DTI FA	*x, y, z*; cluster size; *p*
Soloff	2012	MRI 1,5T	BPD 44/24/0	30/26/26	82/67/46	VBM	*x, y, z*; cluster size; *p*

*SA, suicide attempters; PC, psychiatric controls; HC, healthy controls; S, schizophrenia; MD, major depression; BD, bipolar disorder; BPD, borderline personality disorder; VBM, voxel-based morphometry; DTI, diffusion tensor imaging; FA, fractional anisotropy; SB, suicidal behaviour; BA, Brodmann area; *x, y, z,* stereotactic coordinates.*

**FIGURE 1:**
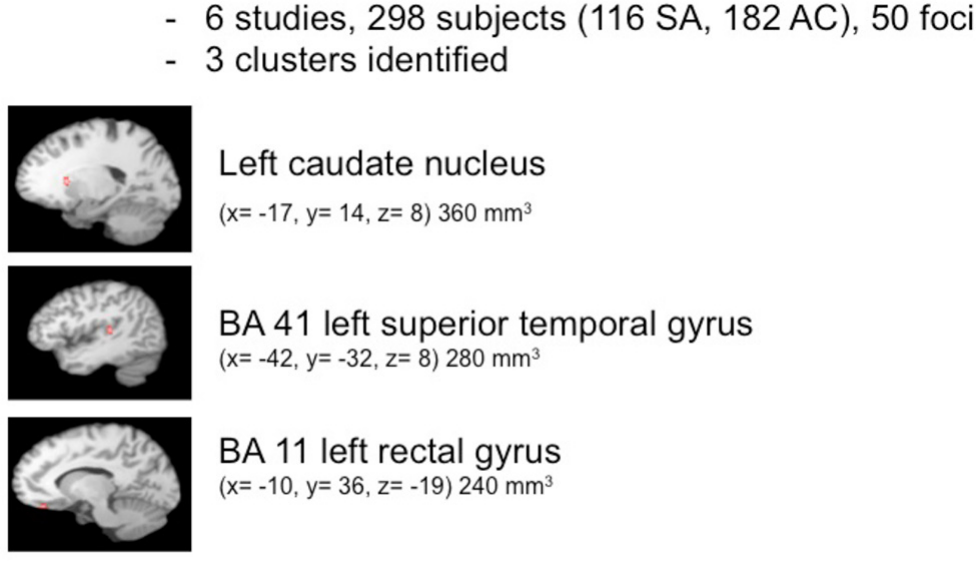
**Meta-analysis of structural imaging studies**.

The pooled study population of the six structural imaging studies in the meta-analysis consisted of 298 individuals, i.e., 116 suicide attempters, and 182 psychiatric controls. The total number of foci identified in the studies was 50. As shown in **Figure 1**, ALE analysis identified three clusters indicating reduced gray matter volume in the left hemisphere, i.e., in the caudate nucleus (cluster size: 360 mm^3^; center: *x* = -16,75, *y* = 13,77, *z* = 8,46), the superior temporal gyrus (cluster size: 280 mm^3^; center: *x* = -41,49, *y* = -31,9, *z* = 8,14), and the rectal gyrus (cluster size: 240 mm^3^; center: *x* = -9,93, *y*= 35,81, *z* = -19,08).

### FUNCTIONAL IMAGING STUDIES

As shown in **Table 2**, six studies were included in the meta-analysis (Jollant et al., 2008, 2010; Pan et al., 2011, 2013a,b; Fan et al., 2013).

**Table 2:** Functional imaging studies included in the meta-analysis.

Study (first author)	Year	Imaging modality	Study population SA/PC/HC	Mean age years	% *F*	Measurement	Available data
Jollant	2008	fMRI 1,5T	MD 13/14/16	40/44/32	0/0/0	facial emotion recognition	BA; *x, y, z*; volume (voxels); *p*
Jollant	2010	fMRI 1,5T	MD 13/12/-	38/43/30	0/0/0	IGT	BA; *x, y, z*; U; *p*
Pan	2011	fMRI 3T	MD 15/15/14	16/16/15	73/53/43	go/no-go	BA; *x, y, z*; F; Z; k (cluster); p
Pan	2013a	fMRI 3T	MD 15/14/13	16/16/15	73/50/38	IGT	BA; *x, y, z*; F; Z; k (cluster); p
Fan	2013	fMRI 3T	MD 27/10/57	34/38/37	59/56/55	ALFF	BA; *x, y, z*; K (cluster number); t
Pan	2013b	fMRI 3T	MD 14/15/15	16/16/15	71/53/47	angry & happy faces	BA; *x, y, z*; F; Z; k (cluster); t; *p*

*SA, suicide attempters; PC, psychiatric controls; HC, healthy controls; MD, major depression; IGT, iowa gambling task; ALFF, amplitude of low-frequency fluctuation; BA, Brodmann area; *x, y, z*, stereotactic coordinates.*

**FIGURE 2:**
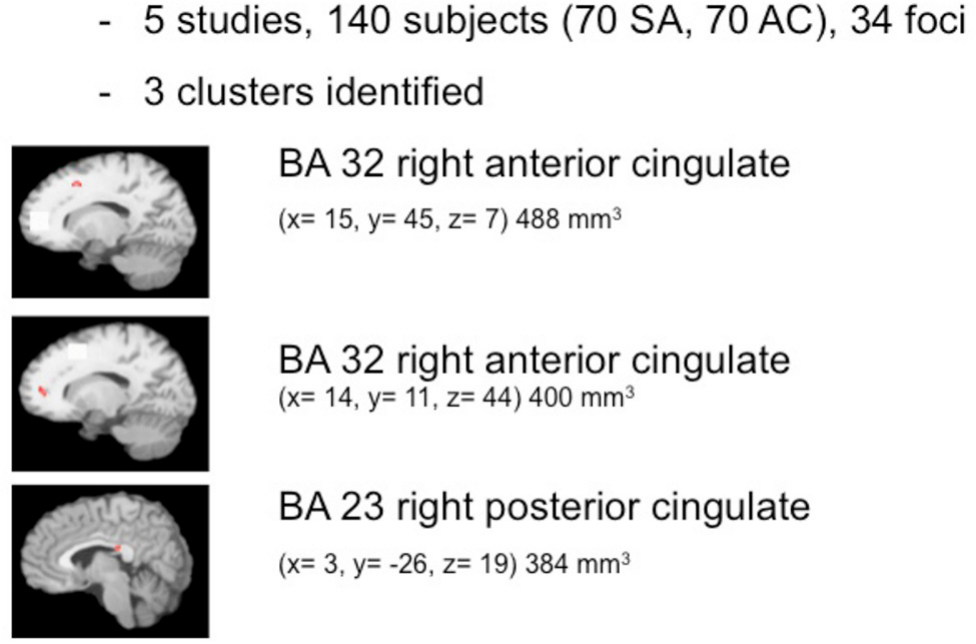
**Meta-analysis of functional imaging studies**.

The pooled study population of the included functional imaging studies in the meta-analysis consisted of 177 individuals, i.e., 97 suicide attempters, and 80 psychiatric controls. The number of foci identified in the studies was 34. As shown in **Figure 2**, ALE analysis identified three clusters in the right hemisphere, i.e., two clusters in the right anterior cingulate and one in the posterior cingulate. Characteristics of the clusters in the right anterior cingulate were (1) cluster size: 480 mm^3^; center: *x*= 14,42, *y* = 44,92, *z* = 7,28 (dorsal anterior cingulate), and (2) cluster size: 400 mm^3^; center: *x*= 13,73, *y* = 11,28, *z* = 43,55 (rostral anterior cingulate). The size of the cluster in the right posterior cingulate was 384 mm^3^, centered at *x* = 2,97, *y* = -25,94, *z* = 19,39. The cluster in the dorsal anterior cingulate showed increased activation in suicide attempters when compared to psychiatric controls during exposure to angry faces or mild happy faces, while activation was relatively less increased in attempters than in psychiatric controls during high-risk decisions. The cluster in the rostral anterior cingulate showed increased activation in suicide attempters when compared to psychiatric controls during exposure to angry faces, while activation was relatively less increased in attempters than in psychiatric controls during a go/no-go task. Finally, the cluster in the posterior cingulate showed increased activation in suicide attempters when compared to psychiatric controls during exposure to happy faces, while activation was relatively less increased in attempters than in psychiatric controls during high-risk decisions.

## DISCUSSION

This is the first meta-analysis of imaging studies of suicidal behavior. We were particularly interested in studying brain correlates of a vulnerability for suicidal behavior, and therefore included only studies, which reported a comparison between individuals with a history of a psychiatric disorder and a history of suicide attempts to individuals with a history of only this psychiatric disorder.

The findings support the existence of a vulnerability to suicidal behavior and suggest a neuroanatomical basis for this diathesis. In summary, ALE meta-analyses of 12 studies identifies six regions in the brain that in association with a history of suicidal behavior are characterized by decreased volumes or changes in reactivity to emotional and cognitive stimuli. The structural correlate of the vulnerability consisted of clusters of smaller volumes in the left superior temporal gyrus, rectal gyrus, and caudate nucleus. Functional correlates of the vulnerability to suicidal behavior were confined to the right cingulate gyrus, with two clusters in the anterior cingulate and one in the posterior cingulate.

Preceding a discussion of these findings a number of methodological issues need to be addressed. The number of studies, which could be included in the meta-analyses, is rather small. Due to methodological and ethical difficulties, inclusion of suitable suicidal patients in neurobiological research is a difficult process, so that it will take a substantial amount of time before a larger number of studies will be available for meta-analyses. The inclusion of a limited number of functional imaging studies implies that the effects of only a small number of activation paradigms have been investigated in suicide attempters. Therefore, it cannot be concluded that a diathesis to suicidal behavior is characterized by changed cingulate reactivity to particular facial emotional expressions or cognitive stimuli. Changes in such reactivity may also occur following exposure to other emotional or cognitive stimuli. In addition, it should be noted that studies were variable in terms of applied definitions of suicidal behavior and psychiatric diagnoses in suicide attempters and controls. The neurobiology of suicidal behavior along with depression may differ from that along with schizophrenia, but evidence of a shared underlying vulnerability is increasing (van Heeringen and Mann, 2014). As imaging studies involving elderly suicide attempters and controls were not included in the meta-analysis, the results of the current meta-analyses may not be applicable to the elderly. The conclusion that the demonstrated changes in brain functions and structures reflect a vulnerability to suicidal behavior is based on the assumption that suicide attempters and controls did not differ with regard to state-dependent characteristics. An effect of such state-dependent characteristics, including the use of medication, is most probably limited due to the strict inclusion criteria, but cannot be ruled out completely. Even with the strict inclusion criteria, the suitable studies still varied on a large number of variables such as statistical thresholds, smoothing kernels, registration procedures to standard space, and MRI scanning parameters. While current coordinate-based meta-analysis methods cannot account for all these differences separately, ALE does estimate a spatial uncertainty per individual study thereby alleviating some of the between-studies variability arising from varying study specific parameters such as the number of subjects or the use of different brain templates (Eickhoff et al., 2009). Finally, disturbances in brain functions may be attributable to structural changes. The differences between structurally and functionally affected regions in the current study, however, suggest that functional changes in association with a diathesis to suicidal behavior are not due to structural disturbances, at least not in the same areas. The relationship between the structural and functional findings will be discussed below.

With regard to the structural findings, combining voxel-based DTI and gray matter data in one meta-analysis may answer fundamental questions such as whether gray and white matter abnormalities, when found, are consistent with one another, and whether white matter alterations are consistent with alterations in gray matter of areas connected by these white matter abnormalities. The current meta-analysis, however, did not identify abnormalities in white matter in association with a history of suicidal behavior. A decreased gray matter volume of the orbitofrontal cortex has been shown in several psychiatric disorders, including depression (Wagner et al., 2008) and anxiety disorders (Strawn et al., 2013). Reduced volumes of the superior temporal gyrus have been demonstrated in schizophrenia (Palaniyappan et al., 2012). Reduced caudate volumes have been found in, among others, bulimia nervosa (Amianto et al., 2013), borderline personality disorder (O’Neill et al., 2013) and depression (Ma et al., 2012). These disorders also share a substantially increased suicide risk (Hawton and van Heeringen, 2009).

At first glance, the structural findings from the meta-analysis reflect a complex pattern of changes, and each of the involved regions may serve multiple functions. However, recent research findings point at a converging function from a cognitive neuroscience point of view, i.e., the processing of negative emotions. The superior temporal gyrus (Radua et al., 2010; Kumfor et al., 2013), the rectal gyrus (Schoenbaum et al., 2011; Szatkowska et al., 2011) and the caudate nucleus (Kemp et al., 2013) are involved in emotion processing, particularly with regard to negative emotions as shown in studies of facial emotion perception. The structures identified as structural correlates of the vulnerability to suicidal behavior appear to be particularly involved in the processing of the punishing aspect of salient events and may thus mediate in planning behavior on the basis of negative information.

The putative role of disturbed emotion processing, resulting in the aberrant salience of particular emotional stimuli, in the vulnerability to suicidal behavior is confirmed by the findings from the meta-analysis of functional imaging studies of suicidal behavior. Two clusters with changed activation patterns in association with a history of suicidal behavior were identified in the ACC, which is a structure in the medial prefrontal cortex that comprises several functional subdivisions. Rostral regions of the ACC (rACC) activate during emotional states or during tasks that involve interference from emotional stimuli. In contrast, dorsal regions of the ACC (dACC) activate during tasks that involve interference from non-emotional stimuli. The current meta-analysis shows increased activation during emotional tasks (i.e., exposure to emotional faces) and decreased activation during cognitive tasks [i.e., the iowa gambling task (IGT) and a Go/No-go task, respectively] in the rostral and in the dorsal ACC in association with a history of suicidal behavior. A distinction between rostral/emotional and dorsal/non-emotional is thus not found in this study, which is in keeping with recent models of functional organization of the brain (Lindquist and Barrett, 2012). A third cluster was identified in the PCC, in a similar way showing increased activation upon perception of emotional faces and decreased activation during the IGT.

The mechanism, by means of which reduced volumes of particular brain areas relate to changes in functional cognitive emotional characteristics, is yet unclear. Structural abnormalities may represent a trait factor and lead to functional changes that represent state factors (de Kwaasteniet et al., 2013). However, only very few studies have focused on the association between structural and functional cognitive or emotional alterations in the brain in the context of psychiatric disorders. No such studies exist with regard to suicidal behavior. Wagner et al. (2008) demonstrated a significant negative correlation between gray matter volume in the gyrus rectus and the BOLD signal in the rACC during the Stroop task in depressed individuals. Scheuerecker et al. (2010) reported a similar negative correlation between Brodmann area 11 (i.e., rectal gyrus) volumes and changes in the BOLD signal in, among others, the left caudate nucleus during an emotional face-matching task. The findings suggest that due to decreased gray matter in the orbitofrontal cortex depressed patients are not able to suppress interfering rACC and caudate activity. As the rACC and caudate are considered part of the brain’s default mode network (DMN), the authors suggest that the inability to deactivate this network during cognitive processing is related to structural deficits in the rectal gyrus. Thus, a dysbalance of the orbitofrontal-cingulate network in controlling maladaptive affective responses during cognitive processing is strongly related to structural lesions within this network (Wagner et al., 2008). As the current meta-analysis showed similar functional disturbances in another part of the DMN, i.e., the PCC, the findings suggest that a vulnerability to suicidal behavior is associated with disturbances in an orbitofrontal- cingulate network, characterized by the interrelated inability to control maladaptive responses during cognitive processing and a reduced volume of the rectal gyrus. Grimm et al. (2009) provided further support for a role of reduced task-induced rACC DMN deactivation in suicidal behavior in depressed individuals by reporting a correlation between decreased negative BOLD responses in the DMN during emotion processing and feelings of hopelessness, a major risk factor for suicide. Thus, the current findings suggest that the increased salience of particular negative stimuli and the inability to control maladaptive responses during cognitive processing due to structural deficits are two core characteristics of the vulnerability to suicidal behavior.

Further insight in the nature of the deficits in cognitive processing associated with a vulnerability to suicidal behavior is provided by findings from neuropsychological studies. A recent meta-analysis showed a particular role of deficits in decision-making, verbal fluency and Stroop interference in this respect (Richard-Devantoy et al., 2014). While the currently demonstrated involvement of the ACC in a vulnerability to suicidal behavior may explain the lower Stroop performance, the findings from the current meta-analyses may particularly shed light on the role of deficient decision-making. The caudate, OFC and ACC are implicated in the process of reinforcement-guided decision-making, but appear to make distinctive contributions. In conjunction with the caudate, OFC codes the stimulus that is target of the action in terms of specific reward expectations, thus determining the tendency to action, i.e., to approach or avoid the predictive stimulus. Delay is an important aspect of relevant information for determining reward expectation. While coding of short-term reward expectation occurs in conjunction with the ventral striatum-based reward system, the dorsal striatum including the caudate nucleus appears to be involved in predicting future reward (Tanaka et al., 2004, 2007; Onoda et al., 2011). The dorsal striatum and its connected cortical control network thus enact motivational control over intentional behavior (Harsay et al., 2011). In keeping with this line of reasoning, a reduced caudate volume was recently found associated with increased delay discounting in Parkinson patients (Szamosi et al., 2013). Given the well-documented involvement of serotonin disturbances in suicidal behavior (Mann, 2013), it is of importance to note that striatal reward prediction at different time scales is modulated by the central serotonergic system (Tanaka et al., 2007).

Taken together, the findings from meta-analyses of neuroimaging and neuropsychological studies thus suggest that the vulnerability to suicidal behavior can be defined in terms of a reduced motivational control over the intentional behavioral reaction to salient negative stimuli. The current meta-analyses have identified structural abnormalities that may represent the vulnerability trait factor, leading to functional changes that may represent the state factors. Further study is needed to confirm these findings and explore network and connectivity characteristics of identified changes in neural substrates, the causes of these changes, which may be genetic or acquired, and the effects of treatments such as antidepressants, rTMS, ketamine, and psychotherapy on structural and functional changes.

## ACKNOWLEDGMENT

The authors have neither financial interest in, nor financial support for writing this meta-analysis.

## Conflict of Interest Statement

The authors declare that the research was conducted in the absence of any commercial or financial relationships that could be construed as a potential conflict of interest.
